# Capturing the impact employees have on their coworkers and leaders: a holistic approach on health-specific support behavior from employees

**DOI:** 10.3389/fpsyg.2023.1183862

**Published:** 2023-06-30

**Authors:** Nora Gosch, Eva-Maria Schulte, Simone Kauffeld

**Affiliations:** Division of Industrial, Organizational and Social Psychology, Faculty of Life Sciences, Institute of Psychology, Technische Universität Braunschweig, Braunschweig, Germany

**Keywords:** employee and leader health, StaffCare, SelfCare, PeerCare, LeaderCare, health-specific support behavior

## Abstract

Support is a valuable resource for ensuring employee health in the workplace. However, research on health-specific support behavior (i.e., support specifically targeting concrete health aspects) has only concentrated on either leader behavior (e.g., healthy leadership styles) or support provided by employees for specific health issues (e.g., healthy eating or smoking cessation). Although the importance of employee health has been well established, the examination of a wider range of potential health-specific support behaviors from employees provided for their colleagues and leaders has been neglected. To understand employee health-specific support behavior, we adapted an existing health-oriented leadership questionnaire to cover support for colleagues (PeerCare) and their leaders (LeaderCare). Capturing the employee perspective with a sample of 347 employees, the results confirmed a delineation of health-oriented scales (factor, convergent, and discriminant validity). By testing health-specific support behavior processes at work, the positive effects of PeerCare on general health were demonstrated. Contrary to expectations, existing health effects are outweighed when leaders provide health-specific support behavior to their employees (StaffCare). However, the results imply that the health-specific support behavior practices of different actors reinforce each other: the effects of StaffCare and PeerCare enhance each other, and StaffCare has a strong influence on LeaderCare. Remarkably, SelfCare has a key role in this process. The open questions and implications regarding the effects of the different health-specific support behavior measurements are discussed.

## 1. Introduction

The negative effects of work on employees’ health are well known (e.g., Crawford et al., 2010; Schulte et al., 2021). Nevertheless, demanding work conditions are quite common in the European Union (EU), with data indicating that 33% of EU citizens work at very high speed, 36% work to tight deadlines, and the work pace of 68% depends on “direct demands from … costumers, passengers, pupils, patients, etc.” (Parent-Thirion et al., 2017). Due to these challenging work conditions, 10.3% of respondents in an EU-wide survey reported health-problems that were caused or worsened by their work (Eurostat, 2021). Consequently, sick leave is high in the EU, at 12.3 days per employee per year (World Health Organization, 2020). This equates to a high cost of up to 2.5% of GDP per year (Edwards and Greasley, 2010). There is clearly a great need for action, so it is therefore unsurprising that health has attracted increasing focus in organizational psychology research. Resources have been shown to be an important lever for occupational health-promotion (Karasek, 1979; Hobfoll, 1989; Demerouti et al., 2001) and healthy leadership is an established resource for employee wellbeing (e.g., Grimm et al., 2021; Hauff et al., 2022; Klug et al., 2022). Studies confirm its positive impact on employee health and wellbeing (e.g., Pundt et al., 2014; Jiménez et al., 2017; Vincent-Höper and Stein, 2019). Researchers emphasize, however, that in addition to managerial support, social support from employees is also important – for their colleagues (Chiaburu and Harrison, 2008) as well as for their leaders (Uhl-Bien et al., 2014). Considering challenges leaders face themselves in everyday work life (e.g., Parker and DeCotiis, 1983; Crompton, 2011; Berntson et al., 2012) as well as large guiding margins (Wooldridge et al., 2008), it seems to be especially promising to integrate health-specific support behaviors of employees as additional resources to foster health at work. Therefore, expanding the concept of healthy leadership to include similar health-directed support behaviors of employees for their leaders (LeaderCare) and colleagues (PeerCare) creates a holistic model of health-fostering exchange processes between members of a work group. Drawing on stress theories (Lazarus and Folkman, 1984; Hobfoll, 1989; Demerouti et al., 2001) and social exchange theory (Cropanzano and Mitchell, 2005), this study aims at establishing health-specific support behavior of employees within the team and toward their leader (e.g., addressing health risks at work, offering solutions when someone is stressed, or motivating others to participate in health promotion) as a relevant component of a holistic health support process at work.

For this purpose, the health-oriented leadership model proposed by Pundt et al. (2014) is particularly well suited as it provides a complex understanding of how leader behavior relates to the health of employees (Pundt and Felfe, 2011, 2017; Pundt et al., 2014). The model encompasses different perspectives by including StaffCare (a leader’s concern for their employees’ health issues), leaders’ SelfCare (how leaders take care of themselves), and employees’ SelfCare (employees’ endeavors to improve their health conditions). Building on research that has demonstrated the impact of general social support (i.e., that is not specifically aimed at health issues) on employee wellbeing (e.g., Iwata, 1997; Beehr et al., 2003; Oxenstierna et al., 2005; Xanthopoulou et al., 2008; de Clercq et al., 2020) as well as on initial findings concerning support for other people in carrying out health behaviors such as healthy eating, quitting smoking, and engaging in physical activity (e.g., Sorensen et al., 1998; Sarkar et al., 2016), we argue that PeerCare and LeaderCare are an important expansion to the health-oriented leadership model.

Therefore, this study firstly examines the psychometric quality of PeerCare and LeaderCare, in addition to the well-established health-oriented leadership model. On this basis, it is explored from an employee perspective how the different forms of health-specific support behavior in the workplace depend on each other and how they are related to employee health.

## 2. Developing a holistic approach to employee health-specific support behavior based on the health-oriented leadership model

Stress theories such as conservation of resources theory, the job-demands and resources model or the transactional model of stress (Lazarus and Folkman, 1984; Hobfoll, 1989; Demerouti et al., 2001) have in common that they integrate resources as a key component. They state, that strengthening resources is a valuable strategy to support employees’ health. One resource that has recently become a focus of research and practice is healthy leadership, which encompasses leaders’ behaviors to promote the health of their subordinates (Pundt et al., 2014; Schulte et al., 2018; Yao et al., 2021). In contrast to other social resources (e.g., social support, role clarity, for more examples see also Schaufeli, 2017), healthy leadership styles are characterized by its direct focus on improving the health of the counterpart. Previous research confirms the positive impact of healthy leadership styles on employee health and wellbeing (e.g., Pundt et al., 2014; Jiménez et al., 2017; Vincent-Höper and Stein, 2019). However, not only leaders can provide direct support for others’ health. From clinical settings it is known, that the support for medication adherence and recovery of spouses is positively related to patients’ health behavior (e.g., Zautra et al., 1998; Franks et al., 2006). In the workplace, health-specific support behavior has been already researched in terms of coworker support for health behaviors: Coworker support for healthy eating is associated with a readiness to improve eating behaviors (Sorensen et al., 1998), support from coworkers for physical activity is positively associated with its repeated execution (Sarkar et al., 2016), and coworker support to cease smoking is helpful in quitting smoking if coworkers take part in the same program (van den Brand et al., 2019). In contrast to healthy leadership styles, the examined support behaviors in these studies are limited to one very specific health behavior and are not generalizable.

Furthermore, prior work calls for additional consideration of the influence of team members on their leaders in general (Uhl-Bien et al., 2014; Güntner et al., 2020), and on their health and wellbeing in particular (Nielsen and Taris, 2019). A qualitative study by St-Hilaire et al. (2019) emphasizes that scientific approaches on support from employees for leaders’ health are scarce. They demonstrated that managers have many ideas how employees could support them, but that employees have difficulty naming concrete strategies although they are willing to support their leaders in order to protect their health.

Therefore, this study aims at establishing PeerCare and LeaderCare with reference to concrete behaviors to help fostering such behavior in the work context. Thus, health-specific support behavior is defined as behavior that helps other people protect or improve their health and enhance other peoples’ health behavior. This definition would also subsume healthy leadership styles as one form of health-specific support behavior from the leader directed at the subordinates. It can be seen as a specific kind of health-promotion behavior, but they are not the same as health-promotion encompasses more than direct support-behaviors. At the same time, health-specific support behavior must be distinguished from self-directed health behaviors as they do not target others.

Moreover, in this way, not only the direct health effects of receiving health-specific support behavior can be considered. Considering health-specific support behavior from different perspectives (i.e., provided by the leader, colleague, or subordinate) allows for a holistic view on a resource exchange system. Social exchange theory says that beneficial behavior can trigger reciprocal behavior by the profiteer in dyadic settings (Cropanzano and Mitchell, 2005). Therefore, it is conceivable that health-specific support behavior triggers congruent reciprocal acts: e.g., an employee showing health-specific support behavior for the leader after receiving similar support from the leader or colleagues taking care for each other in terms of health-specific support behavior.

From research about generalized exchange it is known that reciprocity is not limited to dyadic settings but can also involve third parties (Molm, 2010). This means beneficial actions are not reciprocated to the original source which provided health-specific support behavior initially but are forwarded to a third party. So, health-specific support behavior from the leader for an employee could trigger congruent support from that employee for a coworker. If such a system of generalized exchange establishes this could even contribute to the general development of a positive health climate, resulting in strong relations between the different forms of health-specific support.

### 2.1. PeerCare and LeaderCare as new components of the health-oriented leadership model

Healthy leadership styles comprise important support behaviors from the leader with the aim to improve subordinates’ health directly or indirectly. In this regard they already map one form of health-specific support behavior. Therefore, they are a good basis for adaptation with the goal of capturing other health-specific support behavior relationships. Especially, health-oriented leadership by Pundt et al. (2014) addresses different concrete behaviors, which are focused on directly improving others’ health and is based on a well-constructed and elaborated questionnaire. Pundt et al.’s (2014) model comprises three different aspects––leaders’ StaffCare (i.e., leaders’ concern for their employees’ health issues) as well as leaders’ and employees’ SelfCare (i.e., one’s own endeavor to improve individual health conditions) (Pundt et al., 2014; Pundt and Felfe, 2017). These aspects include the same three dimensions––awareness (i.e., noticing stress and its cause in oneself or others), values (i.e., attaching importance to health issues), and behavior (i.e., taking action regarding health issues) (Pundt et al., 2014). Awareness and values are rather cognitive processes, whereas behavior encompasses the concrete actions that concern personal lifestyles, as well as healthy behavior at work.

The positive health effects of health-oriented leadership have been identified in various settings (e.g., Pundt and Felfe, 2011; Pundt et al., 2014; Horstmann, 2018; Klug and Felfe, 2019; Arnold and Rigotti, 2021; Kaluza et al., 2021). In addition, it has been shown for various health outcomes (i.e., such as burnout, irritation, depression, and physical or somatic complaints) that employee SelfCare mediates these health effects of StaffCare (Pundt et al., 2014; Köppe et al., 2018; Santa Maria et al., 2019). Important prerequisites for StaffCare are that leaders are in good health and take care of themselves in the sense of their own SelfCare (Pundt and Felfe, 2011; Klebe et al., 2022; Klug et al., 2022). Also leaders’ demands and resources impact the StaffCare displayed by the leader (Arnold and Rigotti, 2020; Krick et al., 2022; Pischel et al., 2022). Moreover, the positive relation between StaffCare and employee health is even stronger when leader and employee SelfCare are high (Klug et al., 2019). StaffCare also mediates crossover effects from leaders’ exhaustion to employees’ somatic complaints (Köppe et al., 2018) and can buffer effects of job demands on employee health (Krick et al., 2021). Furthermore, the positive effect of StaffCare has been confirmed by experimental designs (Klebe et al., 2021a).

**Figure 1:**
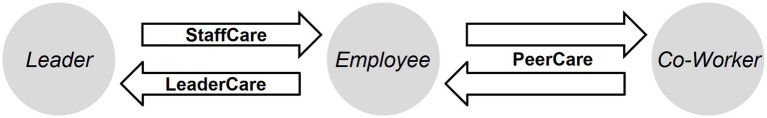
Illustration of the different forms of health-specific support behavior – StaffCare, PeerCare, and LeaderCare.

The empirical results underscore the suitability of the health-focused leadership model as a basis for a holistic model of health-specific support behavior in the workplace. Although employees have already been integrated into this approach, their active contribution is limited to their SelfCare behavior (i.e., receiving support from the leader and taking care of themselves). Changing the perspective on employees as valuable resources at the workplace alongside managers provides the opportunity for a holistic understanding of health-specific support behavior processes in the workplace. To achieve this goal, we first adapted the StaffCare component of the health-oriented leadership questionnaire resulting in two additional health-specific support behavior relations. In reference to the original term StaffCare, the two additional scales are named PeerCare and LeaderCare. PeerCare encompasses health-specific support behavior provided by employees for their coworkers, while LeaderCare captures health-specific support behavior provided by employees for their leader (see Figure 1 for an illustration). After we evaluated the psychometric quality of the two scales, we examined the role of the different forms of health-specific support behavior (PeerCare, LeaderCare, and StaffCare) simultaneously. This allowed for a comparison of the effects of coworkers’ and leaders’ health-specific support behavior (i.e., PeerCare and LeaderCare), while also considering potential crossover processes between them. Potential interrelations of health-specific support behavior by leaders and employees (i.e., StaffCare and LeaderCare) were also investigated. Since the aim of this study is to examine health-specific support behavior, we concentrated on the behavior facet of StaffCare, PeerCare and LeaderCare in this work. Nevertheless, the facets awareness and value were also adapted for PeerCare and LeaderCare.

### 2.2. Construct differentiation of PeerCare, LeaderCare, and StaffCare

StaffCare, PeerCare, and LeaderCare represent different forms of health-specific support behavior in a work team. Although they are very similar regarding concrete behaviors, they differ significantly in terms of the people involved. To promote the health of all team members, all these variants of health-specific support behavior should be present in a team. However, it is conceivable that the different types of health-specific support behavior are not equally available in every team: It might be that supervisors support their group members regarding their health because they see it as their duty, while employees are less supportive of their colleagues or supervisors. Otherwise, it could also be that employees support each other’s health because their supervisor fails to do so, and they feel responsible for each other. There is also the possibility of supervisors and team members being supportive simultaneously, when the supervisor feels responsible for the health of the employees and there is a good health climate in the team. Therefore, in means of factorial validity, the three components––StaffCare, PeerCare, and LeaderCare––are distinguishable from each other.

*H1*: PeerCare, LeaderCare, and StaffCare are distinct factors in employee health-specific support behavior.

The adapted scales (i.e., PeerCare and LeaderCare) and the original scales (i.e., StaffCare and SelfCare) should also be distinguishable in content. The target similarity framework states that employees consider the source of specific acts in social exchange situations and adapt their attitudes and behavior to that source (Lavelle et al., 2007). At the same time, health-specific support behavior is a specific kind of helping behavior that is related to other similar constructs, such as social support. Therefore, StaffCare, PeerCare, and LeaderCare should have close interrelations with equivalent social support measures that portray the same relationships between the individuals involved, but smaller interrelations with other social support relationships. Furthermore, StaffCare, PeerCare, and LeaderCare are characterized by caring for others, whereas SelfCare relates to caring for oneself. Self-concern and other-orientation are two motivational drivers that represent these two tendencies (De Dreu and Nauta, 2009). Being more concerned with oneself and less oriented toward others are associated with higher self-motivation (De Dreu and Nauta, 2009). Therefore, SelfCare should demonstrate a closer relationship with self-concern than with higher orientation toward others. Higher other-orientation is associated with more pro-social motivation (De Dreu and Nauta, 2009), and therefore should be more strongly associated with LeaderCare. Thus, we tested for convergent and discriminant validity with the following hypotheses:

*H2a*: Delimitation of PeerCare and LeaderCare: PeerCare has a stronger correlation with the social support received by colleagues than LeaderCare. LeaderCare has a stronger correlation with the social support provided for the supervisor than PeerCare.

*H2b*: Distinguishing LeaderCare and PeerCare from StaffCare: StaffCare has a stronger correlation with the social support received by the supervisor than LeaderCare or PeerCare. LeaderCare has a stronger correlation with the social support provided for the supervisor than StaffCare. PeerCare has a stronger correlation with the social support received from colleagues than StaffCare.

*H2c*: Distinguishing LeaderCare and PeerCare from SelfCare: SelfCare has a stronger correlation with self-concern than LeaderCare or PeerCare. LeaderCare has a stronger correlation with other-orientation than SelfCare. PeerCare has a stronger correlation with the social support received from colleagues than SelfCare.

### 2.3. The role of PeerCare and LeaderCare in the health-specific support behavior process

**Figure 2:**
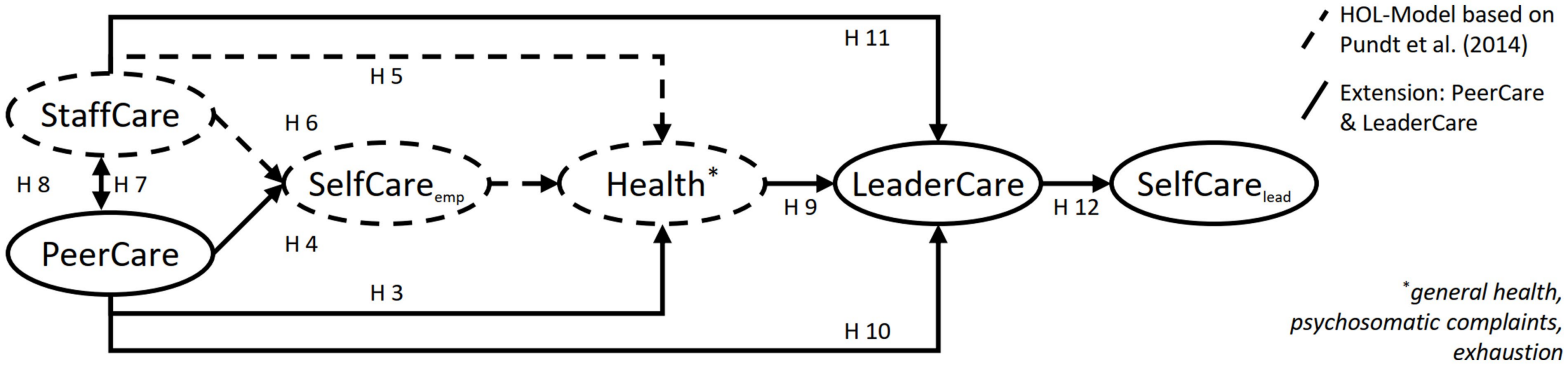
Health-specific support behavior effects and mutual influence processes of health-specific support behaviors from different stakeholders.

In addition to examining the discrimination of PeerCare, LeaderCare, and StaffCare, we aim to develop a holistic health-specific support behavior process which integrates the different relationships (see Figure 2). Criterion and incremental validity are addressed in this context.

As the study’s purpose was to determine ways to enhance employee health at work by capturing employees’ health-specific support behavior, health is one of the most important indicators regarding criterion validity of PeerCare. General social support (Undén, 1996; Borg et al., 2000; Östberg and Lennartsson, 2007; Schmidt et al., 2014) and healthy leadership (Pundt et al., 2014) have a positive impact on general health. In addition, physical complaints are an important health outcome as they are associated with a variety of occupational stressors (Nixon et al., 2011). It has been reported that a group of workers with little support is more likely to have physical complaints than a group with more support (Oxenstierna et al., 2005). Similarly, Pundt et al. (2014) found a negative correlation between StaffCare and health complaints. In the work context, burnout is of special interest as a health indicator. A meta-analysis comparing different sub-dimensions of burnout revealed that social support in the workplace has a stronger negative association with exhaustion, whereas non-work support has a stronger negative association with depersonalization (Halbesleben, 2006). For instance, coworker support reduces exhaustion when controlling for different job demands (Bakker et al., 2005). Therefore, we hypothesize that similar correlations exist between PeerCare and these different health aspects.

*H3*: PeerCare is positively related to (a) general health, (b) somatic complaints, and (c) exhaustion.

However, coworker support not only has a direct impact on health, but has also shown to promote health behaviors such as healthy eating, ceasing smoking, and engaging in physical activity (e.g., Sorensen et al., 1998, 2010; Sarkar et al., 2016), which in turn has a positive impact on health (Pundt et al., 2014). Similar to effects of leaders’ health-specific support behavior for their employees (StaffCare), it was therefore assumed that employees’ health-specific support behavior for their colleagues (PeerCare) would have partially mediating effects on employee health *via* improved SelfCare behavior.

*H4*: PeerCare’s relation with employees’ (a) general health, (b) somatic complaints, and (c) exhaustion is mediated via their own SelfCare.

Furthermore, the relation of StaffCare with employee health has been well documented for different health outcomes like general health, irritation, burnout, depression or psychosomatic complaints (e.g., Pundt et al., 2014; Horstmann, 2018; Klug et al., 2019; Santa Maria et al., 2019). Correspondingly these effects shall be replicated in the scope of this study.

*H5*: StaffCare is positively related with employees’ (a) general health and negatively related with (b) somatic complaints and (c) exhaustion.

Also, the mediation effect of SelfCare for StaffCares’ effect on health outcomes has been demonstrated several times (Pundt et al., 2014; Horstmann, 2018; Santa Maria et al., 2019; Kaluza and Junker, 2022). In order to fully replicate the effect of StaffCare, the mediation effect will also be tested in this study.

*H6*: StaffCare’s relation with employees’ (a) general health, (b) somatic complaints, and (c) exhaustion is mediated *via* their own SelfCare.

Research on indirect reciprocity has indicated that acts of support can also inspire similar behavior in third parties; for example, when the recipient of support transfers the positive experience by supporting someone other than the support provider (Nowak and Sigmund, 2005; Molm, 2010). This can even lead to the development of a generalized exchange system in a group, where acts of help stimulate a virtuous cycle (Simpson et al., 2018). So, employees receiving support from their supervisor might repeat this kind act to a colleague, who might pass on the experience to another colleague. Studies have confirmed similar processes, with vertical leadership positively influencing shared leadership processes in teams (Grille et al., 2015) and health-oriented leadership improving the team’s health climate (Kaluza and Junker, 2022). However, also reverse processes have been discussed, indicating that positive interactions within a team can lead managers to behave positively toward their employees (Zhu et al., 2018; Klasmeier et al., 2022). Leaders who observe positive support dynamics in their team might recognize the importance of such behaviors and be more likely to adopt similar behaviors. Therefore, it can be assumed that the support behaviors regarding health issues of colleagues and leaders enhance each other.

*H7*: PeerCare and StaffCare affect each other positively.

In terms of incremental validity PeerCare should have an additional effect on employee health over StaffCare. For social support, it has been shown that leader and coworker support contribute uniquely to employee health (Luchman and González-Morales, 2013). Therefore, we expected that PeerCare and StaffCare would each make their own contribution to employee health.

*H8*: PeerCare explains additive variance regarding employees’ (a) general health, (b) psychosomatic complaints, and (c) exhaustion, above and beyond StaffCare.

Turning to health-specific support behavior for the supervisor, an important premise might be that employees are healthy and therefore have enough resources to provide helping behaviors. Extra-role behaviors, such as helping (van Dyne and LePine, 1998), are positively correlated with sufficient resources and low demands (Podsakoff et al., 2000). It has been found that sufficient health and resources, along with little demands, are important preconditions for showing extra-role behavior (e.g., Birkeland and Buch, 2015; Costa and Neves, 2017). Similar is also known for leaders to the extent that leaders with low levels of resources and high levels of demands and strain provide less StaffCare for their employees (Klebe et al., 2022; Krick et al., 2022; Pischel et al., 2022). Accordingly, it can be assumed that healthier employees will offer more health-specific support behavior for their supervisor (LeaderCare).

*H9*: The healthier employees are, in terms of (a) general health, (b) somatic complaints, and (c) exhaustion, the more likely they will demonstrate health-specific support behavior for their supervisor (LeaderCare).

The target-similarity approach assumes that employees will adjust their citizenship behavior depending on the target (coworker, supervisor, organization), considering previous experiences with that target in terms of the support and justice experienced (Lavelle et al., 2007). Nevertheless, the framework also allows for weaker cross-sectional and crossover effects between the different levels. A meta-analysis on the effects of coworker support has confirmed the positive effects on organizational citizenship behavior (Chiaburu and Harrison, 2008). Because citizenship behavior includes helping (Podsakoff et al., 2000), we can assume that health-specific support behavior from coworkers may slightly improve health-specific support behavior at other levels, such as for the leader.

*H10*: PeerCare is positively linked to LeaderCare.

Finally, research on reciprocity has shown that receiving help can trigger corresponding behavior––that is, a return of the received favor (e.g., Seinen and Schram, 2006). These patterns can also be observed in supervisor–employee relationships (e.g., Buunk et al., 1993; Scott and Zweig, 2021; Afshan et al., 2022). So, similar interaction patterns can be assumed to occur in a work group engaged in health-specific support behavior. When supervisors engage in more health-specific support behaviors, it is more likely that employees will engage in similar behaviors for their supervisors.

*H11*: The more StaffCare employees receive, the more LeaderCare they will demonstrate.

To support the criterion validity of LeaderCare, we examined its impact on leaders’ SelfCare behaviors. Building on findings that demonstrated that StaffCare is related to employees’ SelfCare behaviors (Pundt et al., 2014; Kaluza et al., 2021; Klebe et al., 2021b; Kaluza and Junker, 2022; Klug et al., 2022), we assumed a positive link between LeaderCare and leaders’ SelfCare behavior. Initial results supported the assumption that employees’ workload and physical strain impact leaders’ workload and physical symptoms (Pindek et al., 2020). Additionally, the findings also demonstrated that support from others can lead to enhanced health behaviors (Sorensen et al., 1998; Gallant, 2003; Graven and Grant, 2014; Sarkar et al., 2016). Building on these examples of crossover effects, we assumed the following hypothesis:

*H12*: LeaderCare is positively linked to leaders’ own health behavior (i.e., SelfCare).

## 3. Materials and methods

### 3.1. Data collection and samples

Data for all relevant scales were collected *via* the German SosciPanel (*n* = 307). This sample was used to calculate correlations, regressions, and path analyses. Due to values missing not at random for the scales StaffCare, LeaderCare, and/or PeerCare (because participants were not working in a team or had no supervisor), only a sub-sample of *n* = 259 could be used for factor analysis (Set-ESEM). Following the procedure described by Hancock and Freeman (2001), 300 participants were targeted as an appropriate sample size for the Set-ESEM. To reach this sample size, a second sub-sample was collected *via* personal approach (*n* = 40), which only included the necessary scales of StaffCare, LeaderCare, and PeerCare. The final sample for factor analysis comprised 299 participants.

Most participants were male (60.8, 34.4% female, 4.7% diverse or unspecified). The mean age was 45.87 (*SD* = 11.22) years and on average they spent 37.26 (*SD* = 10.09) hours working per week. The participants had been working for 12.68 (*SD* = 11.65) years in their current organization.

### 3.2. Measures

#### 3.2.1. PeerCare and LeaderCare

PeerCare and LeaderCare items were generated based on items from StaffCare’s behavior component of the health-oriented leadership scale (Pundt and Felfe, 2017). They were rephrased to refer either to one’s colleagues (PeerCare) or to one’s supervisor (LeaderCare). Six experts rated the content validity of the items in a workshop setting. As a result, the wording of single items was reformulated to realistically reflect the interaction patterns with supervisors or peers, such that the original item “By *making improvements* in the area of working time, *I ensure* that the workload of my employees is reduced …” (Pundt and Felfe, 2017) was reformulated for PeerCare to “In the area of working time, *my colleagues support me* to reduce my workload ….” Since the influence of employees on leaders is limited, the wording for LeaderCare was further toned down to “In the area of working time, *I show my manager ways* to reduce the workload ….” Employees evaluated the received health-specific support behavior from colleagues in order to measure PeerCare (*α* = 0.94). For LeaderCare (*α* = 0.92), the participants evaluated their own behavior toward their supervisors. Both encompassed three items to measure the aspect “personal lifestyle” (α*_PeerCare_* = 0.88; α*_LeaderCare_* = 0.84) and 10 items to measure “behavior at work” (α*_PeerCare_* = 0.93; α*_LeaderCare_* = 0.91). PeerCare and LeaderCare were both measured on a 5-point scale ranging from 1 (*not at all true*) to 5 (*completely true*).

#### 3.2.2. Other measures

Health-oriented leadership was assessed using the behavior dimension of the questionnaire by Pundt and Felfe (2017). StaffCare was measured via 13 items (personal lifestyle: three items, behavior at work: 10 items), with an example item being “By making improvements in the area of working time, my supervisor ensures that my workload is reduced ….” The participant’s SelfCare was assessed using 10 items (e.g., “I make sure that I get enough relaxation and rest”). As this study focuses the behavioral level for StaffCare, PeerCare, and LeaderCare, also employee SelfCare was measured in terms of behavior to maintain the same level of observation. The SelfCare of leaders was measured *via* five items relating to health-risking aspects (e.g., “My manager often does not realize until it’s too late that she’s taken on too much”), which were recoded afterwards. The reliability in this study was *α* = 0.93 for StaffCare (personal lifestyle: *α* = 0.86, work at behavior: *α* = 0.93) and *α* = 0.80 for SelfCare (personal lifestyle: *α* = 0.67, work at behavior: *α* = 0.72). Both SelfCare measures were rated on a 5-point scale ranging from 1 (*not at all true*) to 5 (*completely true*).

Self-concern and other-orientation were measured using three items each (De Dreu and Nauta, 2009). One example item for self-concern is “At work I am concerned about my own needs and interests,” and for other-orientation “At work I am concerned about the needs and interests of others such as my colleagues.” The reliabilities in this study were *α* = 0.76 (self-concern) and *α* = 0.83 (other-orientation). Received and provided social support were indicated *via* four items from Haslam et al. (2018). The items were reworded, as indicated by the authors, to assess support received from the supervisors and colleagues, as well as support provided by the supervisor. Example items are “Do you get the emotional support you need from your leader?” and “Do you give your colleagues the emotional support they need?.” The internal reliability in this study for support received from the supervisor was *α* = 0.92, for support received from colleagues it was *α* = 0.90, and for support provided by the supervisor it was *α* = 0.85. Self-concern and other-orientation were both rated on a 5-point scale ranging from 1 (*not at all true*) to 5 (*completely true*).

Psychosomatic complaints were measured using five items taken from Mohr (1986). One example is “Do you have a sensitive stomach?.” Items were rated on a 5-point scale ranging from 1 (*never or almost never*) to 5 (*almost always*). The reliability in this study was *α* = 0.64. Work-related exhaustion was assessed using the exhaustion sub-scale (eight items) from the Burnout Assessment Tool (Schaufeli and Desart, 2019). This was measured with items like “At work, I feel mentally exhausted” on a 5-point scale ranging from 1 (*never*) to 5 (*always*). The internal consistency measured in this study was *α* = 0.90. The general health condition was measured by an item from the Copenhagen Psychosocial Questionnaire (Kristensen et al., 2005), taken from the German version (Nübling et al., 2005), asking participants to rate their current state of health on a 11-point scale ranging from 0 (*worst conceivable state of health*) to 10 (*best conceivable state of health*). All English scales were translated into German, following the suggestion of Brislin (1970).

### 3.3. Statistical analysis

**Figure 3:**
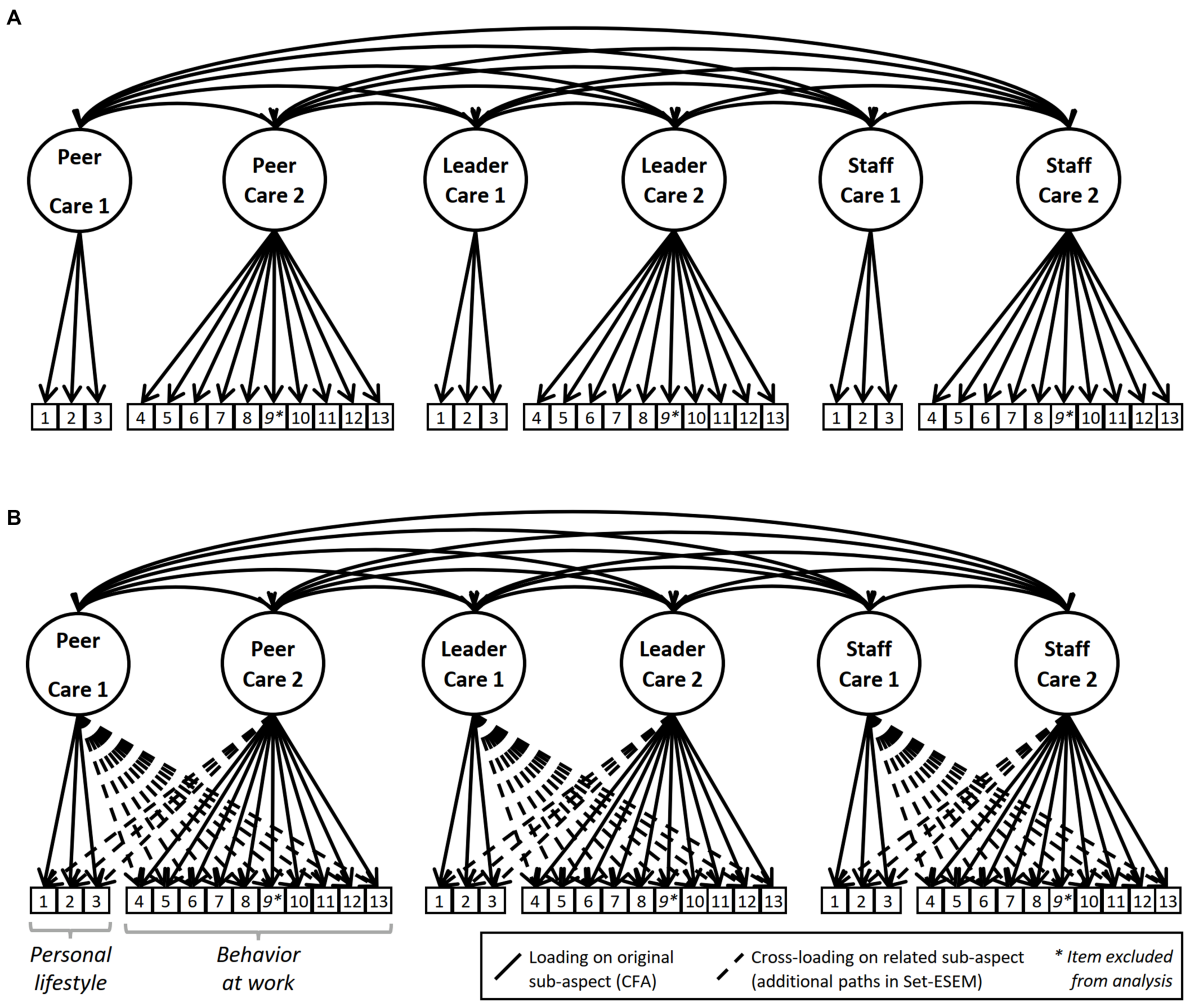
Six-factor model with cross-loadings constrained to 0 (CFA, Panel **A**) and six-factor model with partially freed cross-loadings (Set-ESEM, Panel **B**) used to test factorial validity.

To test for factorial validation (Hypothesis 1), confirmatory factor analysis (CFA) and exploratory structural equation modeling with sets (Set-ESEM) was calculated following the suggestions by Marsh et al. (2020). For this purpose, we used the software Mplus version 8.4 (Muthén and Muthén, 1998–2017) by employing maximum likelihood estimation with robust standard errors. Whereas a CFA allows no cross-loadings between the manifest and latent variables at all, in a Set-ESEM, a specific set of cross-loadings can be defined (Marsh et al., 2020; for a guide, see also van Zyl and ten Klooster, 2021). Accordingly, the two aspects “healthy lifestyle” and “behavior at work” can be considered as related but distinct constructs in a Set-ESEM resulting in six factors with two factors for each scale (PeerCare 1 and 2, LeaderCare 1 and 2, StaffCare 1 and 2). Therefore, two models with six factors were tested for factorial validation: a CFA with cross-loadings constrained to 0 (see Figure 3A) and a Set-ESEM with partly free estimated cross-loadings (see Figure 3B). The chi-squared statistics, root mean square error of approximation (RMSEA), comparative fit index (CFI), and standardized root mean squared residual (SRMR) are reported, and the model fit was estimated using the cut-off values from Schweizer (2010). To account for missing values, a full-information maximum likelihood estimation was used (Enders and Bandalos, 2001).

For convergent and discriminant validity (Hypotheses 2a–c), Pearson correlations were calculated using SPSS software (IBM Corp, 2017). The convergent and discriminant validity were gauged using a *Z*-test to evaluate the difference between two dependent correlations with one variable in common using a tool by Lee and Preacher (2013). To meet the tool’s requirement for a uniform sample size, listwise deletion was chosen for all correlations used to evaluate the convergent and discriminant validation.

A path analysis was performed using Mplus version 8.4 (Muthén and Muthén, 1998–2017) in order to test for the mutual influence processes of health-specific support behavior (Hypotheses 4–11) and the criterion validity of PeerCare (Hypotheses 3a–c) and LeaderCare (Hypothesis 12). The path analysis was conducted in five steps. Model fit and standardized as well as non-standardized covariances are presented for each step. In testing Hypotheses 3a–c, the standardized coefficients correspond to a Pearson correlation (Rodgers and Nicewander, 1988).

## 4. Results

The means, standard deviations, and correlations are given in Table 1. Hypothesis 1 assumed that PeerCare, LeaderCare, and StaffCare can be distinguished in different factors. The tested six-factor structure, as visualized in Figure 3, demonstrated insufficient fit for the CFA (Model A1) and the Set-ESEM (Model B1) with a CFI’s of less than 0.90 (see Table 2). Analysis of the modification indices showed a misfit for Item 9 concerning all examined scales. This item refers to workplace health promotion offers (e.g., from PeerCare: “My colleagues motivate me to take advantage of workplace health promotion offers …”). In an open answer field, participants mentioned several times that their workplace does not offer health promotion measures. The reason why this item distorted the results might be that they did not have the possibility to indicate that a specific item did not apply to their workplace. Therefore, Item 9 was excluded from further analyses for all three scales. In contrast to the adjusted model for the CFA (Model A2), the adjusted Set-ESEM (Model B2) revealed an acceptable fit (see Table 2). Thereby, the adjusted Set-ESEM confirms the assumed factor structure. The factor loadings for the manifest variables corresponding to the assumed sub-scales (i.e., personal lifestyle and behavior at work) ranged from 0.520 to 0.921, whereas the cross-loadings were clearly smaller, with values varying between −0.199 and 0.350 (see Table 3). In addition, the factor correlations between the sub-aspects PeerCare, LeaderCare, and StaffCare corresponded to large effects, with values ranging from *r* = 0.59 to *r* = 0.75 (see Table 4). In summary, Hypothesis 1 was supported.

**Table 1:** Pair-wise correlation matrix with two-tailed significance tests.

	Construct	M (SD)	1	2	3	4	5	6	7	8	9	10	11	12
1	PeerCare	2.69 (0.91)												
2	LeaderCare	2.30 (0.92)	0.37^**^											
3	StaffCare	2.50 (0.95)	0.40^**^	0.57^**^										
4	SelfCare	3.55 (0.61)	0.18^**^	0.24^**^	0.28^**^									
5	Self-Concern	3.32 (0.88)	0.13^*^	0.00	0.10	0.33^**^								
6	Other-orientation	3.63 (0.78)	0.35^**^	0.29^**^	0.21^**^	0.10	0.25^**^							
7	Provided Social Support for Supervisor	4.70 (1.40)	0.23^**^	0.46^**^	0.46^**^	0.21^**^	0.14^*^	0.34^**^						
8	Received Social Support from Supervisor	4.38 (1.67)	0.19^**^	0.39^**^	0.68^**^	0.20^**^	0.11	0.22^**^	0.60^**^					
9	Received Social Support from Colleagues	5.02 (1.32)	0.56^**^	0.17^**^	0.35^**^	0.23^**^	0.23^**^	0.34^**^	0.40^**^	0.43^**^				
10	General Health	6.87 (1.88)	0.18^**^	0.25^**^	0.24^**^	0.42^**^	0.20^**^	0.10	0.32^**^	0.33^**^	0.24^**^			
11	Psychosomatic complaints	2.30 (0.81)	−0.08	−0.10	−0.15^*^	−0.30^**^	−0.15^*^	0.06	−0.15^*^	−0.20^**^	−0.18^**^	−0.55^**^		
12	Exhaustion	2.55 (0.77)	−0.08	−0.16^**^	−0.27^**^	−0.27^**^	−0.12^*^	0.03	−0.26^**^	−0.35^**^	−0.16^**^	−0.51^**^	0.62^**^	
13	SelfCare Supervisor	2.86 (1.02)	0.04	−0.14^*^	0.21^**^	0.06	0.08	0.10	0.12^+^	0.16^**^	0.13^*^	0.06	−0.09	−0.23^**^

*N* = 239–299. ^+^*p* < 0.10, ^*^p < 0.05, ^**^p < 0.01.

**Table 2:** Results for model-fit of CFA and Set-ESEM.

Model		*df*	χ^2^	χ^2^/*df*	RMSEA	CFI	SRMR
Model A1	CFA	687	1,699.80	2.47	0.070	0.86	0.067
Model A2	CFA (*Item 9 excluded*)	579	1,321.02	2.28	0.065	0.89	0.061
Model B1	Set-ESEM	654	1,577.66	2.41	0.069	0.87	0.061
Model B2	Set-ESEM (*Item 9 excluded*)	549	1,203.17	2.19	0.063	0.90	0.054

*N* = 299.

**Table 3:** Standardized factor loadings for manifest variables of PeerCare, LeaderCare, and StaffCare.

Item-Number	PeerCare		LeaderCare		StaffCare	
	PeerCare 1	PeerCare 2	LeaderCare 1	LeaderCare 2	StaffCare 1	StaffCare 2
Personal lifestyle						
01	0.921^**^	−0.045	0.845^**^	−0.009	0.895^**^	−0.016
02	0.848^**^	0.032	0.876^**^	0.042	0.836^**^	0.094
03	0.760^**^	0.013	0.661^**^	0.009	0.568^**^	0.180
Behavior at work						
04	0.163^*^	0.707^**^	0.078	0.792^**^	0.142^*^	0.660^**^
05	−0.009	0.854^**^	−0.199	0.984^**^	−0.008	0.848^**^
06	0.204^*^	0.577^**^	0.181	0.520^**^	0.214^*^	0.560^**^
07	0.003	0.821^**^	−0.002	0.735^**^	0.051	0.751^**^
08	0.350^**^	0.558^**^	0.287^**^	0.584^**^	0.322^**^	0.554^**^
09	*Excluded from analysis*					
10	0.018	0.812^**^	0.172	0.538^**^	−0.083	0.889^**^
11	0.205^*^	0.577^**^	0.305^+^	0.528^**^	0.094	0.687^**^
12	−0.075	0.805^**^	0.132	0.669^**^	−0.104	0.821^**^
13	−0.122	0.762^**^	−0.138	0.660^**^	0.007	0.748^**^

*N* = 299. ^+^*p* < 0.10, ^*^p < 0.05, ^**^p < 0.01. Factor loadings of manifest variables matching the corresponding sub-scale (personal lifestyle and behavior at work) are printed in bold.

**Table 4:** Factor correlations for the six tested factors.

	Factor	1	2	3	4	5
1	PeerCare 1					
2	PeerCare 2	0.66^**^				
3	LeaderCare 1	0.25^**^	0.24^**^			
4	LeaderCare 2	0.29^**^	0.43^**^	0.75^**^		
5	StaffCare 1	0.28^**^	0.36^**^	0.50^**^	0.56^**^	
6	StaffCare 2	0.16^*^	0.44^**^	0.33^**^	0.61^**^	0.59^**^

*N* = 299. ^*^p < 0.05, ^**^p < 0.01.

Although the Set-ESEM results supported the distinction between personal lifestyle and workplace behavior for PeerCare, LeaderCare, and StaffCare, differences for these sub-scales in the following validation hypotheses were not expected. Nevertheless, we calculated all the following analyses for both the aggregated values of PeerCare, LeaderCare, and StaffCare and their sub-scales. However, the separate results for personal lifestyle and workplace behavior were only reported if they differed from the aggregated results.

To account for convergent and discriminant validity, Hypotheses 2a–c suppose that PeerCare, LeaderCare, and StaffCare can be delimitated from one another in terms of their relations with the constructs social support, self-concern and other-orientation. Concerning delimitation of PeerCare and LeaderCare (Hypothesis 2a) results support convergent and discriminant validity (see also Table 5): *Z*-tests support the assumed differentiation for “provided social support for supervisor” (*r_LeaderCare_* = 0.46, *r_PeerCare_* = 0.23, *Z* = 3.50, *p* < 0.001) and “received social support from colleagues” (*r_LeaderCare_* = 0.18, *r_PeerCare_* = 0.56, *Z* = −6.01, *p* < 0.001). Delimitation of PeerCare and StaffCare (Hypothesis 2b) was supported as well regarding “received social support from colleagues” (*r_PeerCare_* = 0.56, *r_StaffCare_* = 0.35, *Z* = 3.52, *p* < 0.001) and “received social support from supervisor” (*r_PeerCare_* = 0.20, *r_StaffCare_* = 0.68, *Z* = −8.45, *p* < 0.001). The second part of Hypothesis 2b, delimitation of LeaderCare and StaffCare, could just be confirmed partially: delimitation worked for “received social support from supervisor” (*r_LeaderCare_* = 0.39, *r_StaffCare_* = 0.68, *Z* = −6.25, *p* < 0.001) but not for “received social support for supervisor” (*r_LeaderCare_* = 0.46, *r_StaffCare_* = 0.48, *Z* = −0.39, *n.s.*) due to the almost equal correlation sizes. Delimitation of SelfCare (Hypothesis 2c) could be supported for PeerCare (received social support from colleagues: *r_PeerCare_* = 0.56, *r_SelfCare_* = 0.24, *Z* = 4.46, *p* < 0.001; self-concern: *r_PeerCare_* = 0.15, *r_SelfCare_* = 0.34, *Z* = −2.39, *p* = 0.008) and LeaderCare (other-orientation: *r_LeaderCare_* = 0.29, *r_SelfCare_* = 0.15, *Z* = 1.84, *p* = 0.033; self-concern: *r_LeaderCare_* = 0.00, *r_SelfCare_* = 0.34, *Z* = −4.43, *p* < 0.001). However, for Hypothesis 2c separate analyses revealed deviations for the personal lifestyle aspect: PeerCare and SelfCare could not be differentiated regarding self-concern (*r_PeerCare_* = 0.16, *r_SelfCare_* = 0.20, Z = −0.46, *p* = 0.323) and LeaderCare and SelfCare could not be differentiated regarding other-orientation (*r_LeaderCare_* = 0.20, *r_SelfCare_* = 0.13, Z = 0.96, *p* = 0.168). Therefore, Hypothesis 2c was partially supported.

**Table 5:** Differentiation between PeerCare, LeaderCare, SelfCare, and StaffCare – results for convergent and discriminant validity.

		r		Z
Hypothesis 2a				
	**LeaderCare**	**vs.**	**PeerCare**	
Provided Social Support for Supervisor	0.46^**^		0.23^**^	3.50^**^
Received Social Support from Colleagues	0.18^**^		0.56^**^	−6.01^**^
Hypothesis 2b				
	**PeerCare**	**vs.**	**StaffCare**	
Received Social Support from Colleagues	0.56^**^		0.35^**^	3.52^**^
Received Social Support from Supervisor	0.20^**^		0.68^**^	−8.45^**^
	**LeaderCare**	**vs.**	**StaffCare**	
Received Social Support from Supervisor	0.39^**^		0.68^**^	−6.25^**^
Provided Social Support for Supervisor	0.46^**^		0.48^**^	−0.39
Hypothesis 2c				
	**PeerCare**	**vs.**	**SelfCare**	
Received Social Support from Colleagues	0.56^**^		0.24^**^	4.46^**^
Self-Concern	0.15^**^		0.34^**^	−2.39^**^
	**LeaderCare**	**vs.**	**SelfCare**	
Other-orientation	0.29^**^		0.15^**^	1.84^*^
Self-Concern	0.00		0.34^**^	−4.43^**^

*N* = 243, ^*^p < 0.05, ^**^p < 0.01.

The path analysis to test Hypotheses 3–12 (see Table 6) revealed that the models tested for Step 1–4 were just identified yielding no information about the overall model fit. Model fit of Step 5 was not sufficient (Model 1: *χ*^2^ (22) = 32.74, RMSEA = 0.13, CFI = 0.87; Model 2:: *χ*^2^ (22) = 35.96, RMSEA = 0.14, CFI = 0.83; Model 3:: *χ*^2^ (22) = 41.97, RMSEA = 0.16, CFI = 0.80).

**Table 6:** Model fit and path coefficients for direct and indirect effects of path analysis for different health outcomes.

		Model 1: General Health		Model 2: Psycho-somatic complaints		Model 3: Exhaustion	
		*B*	Beta	*B*	Beta	*B*	Beta
Step 1	Direct effect						
	PeerCare → Health	0.369^**^	0.188^**^	−0.065	−0.077	−0.084^+^	−0.104^+^
Step 2	Direct effect						
	SelfCare_emp_ → Health	1.210^**^	0.396^**^	−0.391^**^	−0.298^**^	−0.329^**^	−0.262^**^
	PeerCare → Health	0.213^+^	0.108^+^	−0.017	−0.020	−0.043	−0.053
	PeerCare → SelfCare_emp_	0.123^**^	0.191^**^	0.123^**^	0.191^**^	0.123^**^	0.191^**^
	Indirect effect						
	PeerCare → SelfCare_emp_ → Health	0.149^**^	0.076^**^	−0.048^*^	−0.057^*^	−0.040^*^	−0.050^*^
Step 3	Direct effect						
	SelfCare_emp_ → Health	1.135^**^	0.369^**^	−0.365^**^	−0.278^**^	−0.274^**^	−0.217^**^
	PeerCare → Health	0.125	0.064	0.011	0.013	0.022	0.028
	StaffCare → Health	0.237^*^	0.120^*^	−0.066	−0.078	−0.169^**^	−0.208^**^
	PeerCare → SelfCare_emp_	0.050	0.079	0.052	0.081	0.053	0.084
	StaffCare → SelfCare_emp_	0.166^**^	0.260^**^	0.163^**^	0.255^**^	0.161^**^	0.251^**^
	PeerCare ↔ StaffCare	0.385^**^	0.426^**^	0.385^**^	0.426^**^	0.381^**^	0.423^**^
	Indirect effect						
	PeerCare → SelfCare_emp_ → Health	0.057	0.029	−0.019	−0.023	−0.015	−0.018
	StaffCare → SelfCare_emp_ → Health	0.189^**^	0.096^**^	−0.060^**^	−0.071^**^	−0.044^**^	−0.054^**^
Step 4	Direct effect						
	Health → LeaderCare	0.055^*^	0.111^*^	−0.019	−0.016	−0.012	0.010
	PeerCare → LeaderCare	0.142^*^	0.147^*^	0.150^*^	0.155^*^	0.149^*^	0.154^*^
	StaffCare → LeaderCare	0.477^**^	0.488^**^	0.497^**^	0.510^**^	0.503^**^	0.514^**^
	SelfCare_emp_ → Health	1.136^**^	0.369^**^	−0.365^**^	−0.287^**^	−0.274^**^	−0.217^**^
	PeerCare → Health	0.123	0.063	0.010	0.012	0.024	0.030
	StaffCare → Health	0.242^*^	0.123^*^	−0.066	−0.078	−0.170^**^	−0.209^**^
	PeerCare → SelfCare_emp_	0.050	0.079	0.052	0.081	0.053	0.083
	StaffCare → SelfCare_emp_	0.166^**^	0.260^**^	0.163^**^	0.255^**^	0.161^**^	0.251^**^
	PeerCare ↔ StaffCare	0.387^**^	0.428^**^	0.387^**^	0.428^**^	0.383^**^	0.425^**^
	Indirect effect						
	PeerCare → SelfCare_emp_ → Health	0.057	0.029	−0.019	−0.023	−0.015	−0.018
	StaffCare → SelfCare_emp_ → Health	0.189^**^	0.096^**^	−0.060^**^	−0.071^**^	−0.044^**^	−0.055^**^
Step 5	Direct effect						
	Health → LeaderCare	0.055^*^	0.110^*^	*−0.018*	*−0.015*	*0.009*	*0.007*
	PeerCare → LeaderCare	0.160^*^	0.158^*^	0.168^*^	0.166^*^	0.168^*^	0.166^*^
	StaffCare → LeaderCare	0.477^**^	0.488^**^	0.497^**^	0.510^**^	0.502^**^	0.514^**^
	SelfCare_emp_ → Health	1.137^**^	0.369^**^	−0.364^**^	−0.277^**^	−0.275^**^	−0.218^**^
	PeerCare → Health	*0.122*	*0.060*	*0.001*	*0.001*	*0.037*	*0.044*
	StaffCare → Health	0.245^*^	0.124^*^	*−0.063*	*−0.075*	−0.175^**^	−0.209^**^
	PeerCare → SelfCare_emp_	*0.054*	*0.081*	*0.055*	*0.083*	*0.057*	*0.085*
	StaffCare → SelfCare_emp_	0.167^**^	0.261^**^	0.163^**^	0.255^**^	0.161^**^	0.252^**^
	PeerCare ↔ StaffCare	0.350^**^	0.404^**^	0.350^**^	0.404^**^	0.347^**^	0.402^**^
	LeaderCare → SelfCare_lead_	−0.158^*^	−0.144^*^	−0.158^*^	−0.144^*^	−0.158^*^	−0.144^*^
	Indirect effect						
	PeerCare → SelfCare_emp_ → Health	*0.061*	*0.030*	*−0.020*	*−0.023*	*−0.016*	*−0.019*
	StaffCare → SelfCare_emp_ → Health	0.189^**^	0.096^**^	−0.059^**^	−0.071^**^	−0.044^**^	−0.055^**^

N_step1-2_ = 285–299, N_step3-5_ = 307. ^+^*p* < 0.10, ^*^p < 0.05, ^**^p < 0.01. Values in italics indicate insufficient model fit.

In Step 1 of the path analysis criterion validity of PeerCare was addressed with Hypothesis 3 assuming a positive relation with general health and negative relations with somatic complaints and exhaustion (for results see Table 6). Results revealed a positive correlation of PeerCare with general health (*r* = 0.188, *p* = 0.003). The correlation with psychosomatic health complaints was not significant (*r* = −0.077, *n.s*.). For exhaustion, the correlation was marginally significant (*r* = −0.104, *p* = 0.087), but a separate analysis revealed a difference––the “behavior at work” aspect demonstrated a clear negative correlation with exhaustion (*r* = −0.118, *p* = 0.044), but “personal lifestyle” did not (*r* = −0.014, n.s.). Therefore, PeerCare’s criterion validity was supported in terms of general health (Hypothesis 3a) and partly for exhaustion (Hypothesis 3c) but was not supported for psychosomatic complaints (Hypothesis 3b).

Hypothesis 4 assumed that PeerCare’s relation with employees’ (a) general health, (b) somatic complaints, and (c) exhaustion is mediated *via* their own SelfCare. However, testing the health-specific support behavior model in Step 2 (see Table 6) revealed that PeerCare no longer had a direct effect on the different health outcomes when SelfCare was considered at the same time (Model 1: *B* = 0.213, *p* = 0.075; Model 2: *B* = −0.017, *p* = 0.738; Model 3: *B* = −0.043, *p* = 0.377) but a positive impact on SelfCare in all three models (Models 1–3: *B* = 0.123, *p* = 0.002). SelfCare in turn had a clear effect on health outcomes (Model 1: *B* = 1.210, *p* < 0.001; Model 2: *B* = −0.391, *p* < 0.001; Model 3: *B* = −0.329, *p* < 0.001). Correspondingly, SelfCare was found to mediate the effect of PeerCare on general health (*B* = 0.149, *p* = 0.006), psychosomatic complaints (*B* = −0.048, *p* = 0.010), and exhaustion (*B* = −0.040, *p* = 0.013). Thus, Hypotheses 4a–c were supported, as PeerCare influenced employees’ health indirectly *via* SelfCare, but not directly on its own.

Hypothesis 5–8 were tested in Step 3 of the path analysis. Hypothesis 5 supposes that StaffCare is positively related with employees’ general health and negatively related with somatic complaints and exhaustion. Model results from step 3 confirm this hypothesis for (a) general health (Model 1: *B* = 0.237, *p* = 0.040) and (c) exhaustion (*B* = −0.169, *p* = 0.003) but not for (b) psychosomatic complaints (*B* = −0.066, *n.s.*).

Hypothesis 6 addresses SelfCare’s mediation effect for StaffCare’s relation with employees’ (a) general health, (b) somatic complaints, and (c) exhaustion. StaffCare’s effect on health outcomes was indeed mediated in the expected direction by SelfCare for general health (direct: *B* = 0.237, *p* = 0.040; indirect: *B* = 0.096, *p* < 0.001), psychosomatic complaints (direct: *B* = −0.066, *p* = 0.251, indirect: *B* = −0.060, *p* = 0.001), and exhaustion (direct: *B* = −0.169, *p* = 0.003; indirect: *B* = −0.044, *p* = 0.005). Therefore, Hypothesis 6a–c was confirmed, due to its own contribution to employee health.

Hypothesis 7 predicted that PeerCare and StaffCare are linked positively. The intercorrelation of PeerCare and StaffCare was supported for all three models (Model 1: *B* = 0.385, *p* < 0.001; Model 2: *B* = 0.385, *p* < 0.001; Model 3: *B* = 0.381, *p* < 0.001), thereby confirming Hypothesis 7.

Concerning Hypothesis 8, it is assumed that PeerCare explains additive variance regarding employees’ general health, psychosomatic complaints, and exhaustion above and beyond StaffCare.

However, when StaffCare was included in the model, PeerCare had no effect on SelfCare (Model 1: *B* = 0.050, *p* = 0.243; Model 2: *B* = 0.052, *p* = 0.231; Model 3: *B* = 0.053, *p* = 0.218), general health (direct: *B* = 0.125, *p* = 0.295; indirect: *B* = 0.057, *p* = 0.258), psychosomatic complaints (direct: *B* = 0.011, *p* = 0.844; indirect: *B* = −0.019, *p* = 0.258), or exhaustion (direct: *B* = 0.022, *p* = 0.653; indirect: *B* = −0.015, *p* = 0.254). Thus, Hypothesis 8 had to be rejected as PeerCare had no unique impact on health alongside StaffCare.

Hypothesis 9–11 were tested with Step 4 of the path analysis. Hypothesis 9 claims that the healthier employees are, in terms of general health, somatic complaints, and exhaustion, the more likely they will demonstrate health-specific support behavior for their supervisor (LeaderCare). This was supported for general health (*B* = 0.055, *p* = 0.038), but not for psychosomatic complaints (*B* = −0.019, *p* = 0.755) or exhaustion (*B* = −0.012, *p* = 0.847). Hypothesis 10 supposes that PeerCare is positively linked to LeaderCare, which was confirmed for all three models (Model 1: *B* = 0.142, *p* = 0.023; Model 2: *B* = 0.150, *p* = 0.017; Model 3: *B* = 0.149, *p* = 0.016). Hypothesis 11 expects that the more StaffCare employees receive, the more LeaderCare they will demonstrate. This was evident in all three models (Model 1: *B* = 0.477, *p* < 0.001; Model 2: *B* = 0.497, *p* < 0.001; Model 3: *B* = 0.503, *p* < 0.001). The remaining model results from the fourth step did not deviate from the results from the third step.

Hypothesis 12 emphasized a positive relation of LeaderCare and leaders’ SelfCare. As the fit of the regarding models in Step 5 was insufficient, interpretation of the path coefficients is not allowed. Descriptively path coefficients reveal a negative relation of LeaderCare with SelfCare of the leader. If we also take into account the results of correlational analysis (Table 1), this relationship is confirmed (*r* = −0.14, *p* = 0.024). Accordingly, the effect was opposite to the hypothesized direction, which speaks against the criterion validity for LeaderCare.

## 5. Discussion

In this study, we introduced a novel approach to organizational research by examining employees’ health-specific support behavior strategies for their coworkers and leaders. We proposed that employees uniquely contribute to their colleagues’ and leaders’ health by providing support specifically directed at their health issues. Although not all our hypotheses were confirmed, the study revealed new insights into employees’ role in the health-specific support behavior process. By adapting leaders’ StaffCare to employee behavior toward their coworkers (PeerCare) and leaders (LeaderCare), a foundation was laid for a holistic model encompassing health-specific support behavior structures in teams.

An analysis of factor validity confirmed structural differentiation of the three aspects of health-specific support behavior at work (i.e., PeerCare, LeaderCare, and StaffCare). In relation to goodness-of-fit the adjusted Set-ESEM solution was preferable to the adjusted CFA, which speaks for the assumed structure of related sub-aspects (i.e., personal lifestyle and behavior at work), although results of the adjusted CFA and Set-ESEM were descriptively rather similar. Regarding their content (convergent and discriminant validity), PeerCare, LeaderCare, and SelfCare were distinguished from each other, as well as PeerCare from StaffCare. This differentiation in terms of structure and content suggests that health-specific support behavior from employees is a meaningful addition to previous research on promoting health at work. The content-related differentiation of LeaderCare and StaffCare was only partially supported because both were equally related to “provided social support for the supervisor.” The relationship between LeaderCare and provided support for the supervisor was in the expected range. However, contrary to expectations, the relationship between StaffCare and provided support for the supervisor was surprisingly large. This suggests that providing support to a supervisor is a behavior that employees just demonstrate when they feel supported and treated well by their supervisor. In this line, StaffCare might be a premise for support behavior of employees regarding their leader. This is supported by meta-analytical findings about leader–member exchange that indicate leaders have a stronger influence on exchange relationships than their followers do (Dulebohn et al., 2012). In addition, the target-similarity framework states that perceived support by the supervisor can lead employees to return such social exchange behavior (Lavelle et al., 2007). In this sense, LeaderCare and general support behavior of employees for their leader is a form of reciprocity, which could explain why the correlation size of support provided to the leader and StaffCare was comparable to that of LeaderCare. Nevertheless, the expected higher correlation between StaffCare and “received social support from the supervisor” supported the differentiation between StaffCare and LeaderCare.

Analyzing the sub-aspects of PeerCare and LeaderCare revealed another exception regarding convergent and discriminant validity. Whereas the analyses of behavior at work supported the expected results, personal lifestyle did not always yield results consistent with the hypotheses. To understand these deviations, it is important to note that the correlating variables all refer to the work context. Possibly, behavior regarding work issues, such as caring for others’ needs at work, did not necessarily correlate with behavior regarding private issues, such as motivating others to pursue a healthy lifestyle in their free time. This is in line with the target-similarity approach, which states that peoples’ behaviors and attitudes depend on the triggering source (Lavelle et al., 2007). This might explain, for example, why the relationship between support for health issues in the workplace and the private context was weaker.

The results for PeerCare’s health effects were mixed. For general health, a direct effect on health from PeerCare was supported in terms of criterion validity, and the size of the relationship was comparable to meta-analytical findings (Viswesvaran et al., 1999). With no health-specific support behavior from the leader (StaffCare), health-specific support behavior from colleagues (PeerCare) had an impact on general health by improving employees’ SelfCare. In contrast to general health, the effect of PeerCare on psychosomatic complaints was mediated solely by SelfCare. In the case of exhaustion, the indirect effect of PeerCare was also confirmed, as well as the aspect of behavior at work, which had a direct effect. One reason for the greater impact of work-related PeerCare might be that exhaustion is measured in the same domain (i.e., the work context). There have been similar findings from work–family–conflict research, with conflict having a greater impact in the same, rather than in the cross-domain (Amstad et al., 2011).

Overall, the effect of StaffCare on the different health outcomes could be confirmed, although it was only an indirect effect via SelfCare for psychosomatic health complaints. In line with previous research (Pundt et al., 2014; Horstmann, 2018; Santa Maria et al., 2019), our results underline the positive impact of StaffCare on health outcomes. However, present health effects of PeerCare disappeared when StaffCare was considered simultaneously. Accordingly, incremental validity is not supported for PeerCare in this study indicating that leaders’ health-specific support behavior is more important than that of the colleagues. This calls into question, whether PeerCare can provide extra information regarding employee health in addition to StaffCare. Thus, this study joins an ongoing debate about the importance of support from colleagues versus supervisors (Hämmig, 2017). Nevertheless, other findings of this work underscore the value of integrating PeerCare into the holistic health promotion model. The confirmed relation between StaffCare and PeerCare as shown in Table 6 indicates mutual reinforcement: e.g., employees who receive help from their leader passing on similar helping behaviors to their colleagues. These observed results can be seen as an indicator of a generalized exchange system of health-specific support behavior acts in work groups, as has been previously suggested (Simpson et al., 2018). Furthermore, the significant results for PeerCare described above indicate that PeerCare might be a buffer when StaffCare is missing. This is particularly promising in light of previous findings by Mayo et al. (2012) who determined that, when a stressor originates in the supervisor, only coworker but not supervisor support can buffer the negative consequences. They also demonstrated that supervisor support is helpful when a stressor is perceived to originate outside of the supervisor’s realm. In these cases, supervisor support is more effective than coworker support. This could be because managers have more scope to reorganize the work in response to such demands than the coworkers have.

An important external indicator with potential health consequences that occurred during our data collection was the coronavirus pandemic, which was especially demanding for people at that time (Xiong et al., 2020). Following the reasoning of Mayo et al. (2012), supervisor support might have been particularly helpful in dealing with health consequences during the pandemic. Additionally, people worked more from home (Kohlrausch and Zucco, 2020), which is characterized by minimized affiliation and social exchange at work (Baruch, 2000). So, the pandemic also resulted in fewer opportunities to provide support. However, leaders might have been able to counteract the negative effects of virtual work, even though it might have taken more effort on their part (Purvanova and Bono, 2009). Support for health issues may not have been enough at this time. When support does not meet the actual needs, it does not reach its potential benefit (Beehr et al., 2010; Melrose et al., 2015).

To summarize, PeerCare’s psychometric quality was supported in terms of content validity and criterion validity regarding general health and exhaustion (the latter only for the behavior at work aspect of PeerCare). Coworkers are particularly influential on each other’s general health. Negative health outcomes, such as exhaustion and psychosomatic health complaints, are more affected by supervisors’ health-specific support behavior. This is surprising, as team health climate has already been found to be an important factor in individual health (Schulz et al., 2017). For future studies, this raises the question of the circumstances in which health-specific support behavior from colleagues is really needed, when support from the supervisor is sufficient, and which other influential factors are important.

Besides the significant effect of the supervisors, also employees’ SelfCare plays a key role in the health-specific support behavior process. The effects of SelfCare are much larger than those of StaffCare or PeerCare. In clinical studies, SelfCare has already been determined as an important premise in health improvement (Gallant, 2003; Graven and Grant, 2014) and results highlight the potential of SelfCare in occupational health research. Because SelfCare seems to have an essential role in individual health status, how SelfCare can be enhanced is of special interest. Possibly, not only the quality of PeerCare is of interest, but also that PeerCare is distributed equally within teams. This approach is already known from research concerning team–member exchange differentiation (Liao et al., 2010), which has been demonstrated as being positively related to self-efficacy. Self-efficacy, in turn, positively influences health promotion behaviors (Sheeran et al., 2016).

Regarding LeaderCare, results from path analysis cannot be used to deduce interpretations for criterion validity. Alternatively looking at correlation analysis, reveals an unexpected negative relation of LeaderCare with supervisors’ SelfCare, thus questioning its criterion validity. A meta-analysis has claimed that, in most situations, support mitigates strain instead of being elicited when strain is high (Viswesvaran et al., 1999). However, as this study assessed leaders’ SelfCare from the employee perspective, it could be that participants were indicating to support their supervisor more especially when they perceived their leader to be more demanded. This may represent a form of a self-serving bias (see Bradley, 1978). Another possibility might be that in the case of leaders, health-specific support behavior from subordinates can rather enhance feelings of strain instead of reducing them. It has been reported that receiving support from subordinates can make supervisors feel inadequate, which rather exacerbates their health problems (Beehr et al., 2010). This would mean, that support dynamics known from other settings cannot be easily transferred to a situation where employees support their supervisor. Given these two possible explanations, it is necessary to capture the leaders’ perspective in order to reach a conclusive evaluation of criterion validity for LeaderCare.

Confirming the particular role of positive health outcomes in the health-specific support behavior process, LeaderCare was only related with employees’ general health, but not with psychosomatic complaints or exhaustion. PeerCare was found to affect LeaderCare, indicating that supportive behaviors in the workplace between team members can crossover to the leader. Similar effects were found in a study in which exchange with the supervisor had an impact not only on the citizenship behavior toward the supervisor, but also toward the organization (Rupp and Cropanzano, 2002). Moreover, the positive effect of StaffCare on LeaderCare suggests that there are reciprocal interactions between leaders and their team members in the workplace. When eliciting reciprocity, it is not just the employees profiting from StaffCare, but also the leaders themselves.

This study also provides practical implications for PeerCare and LeaderCare. Together with StaffCare they can be used as an easy way to make employees aware of their own social resources and to start reflection processes how health-specific support can be provided for others. Furthermore, the mutually reinforcing processes demonstrated in this study suggest that health-specific support behavior might be a good starting point to generate a positive health climate in the workplace. PeerCare and LeaderCare can be also utilized to develop training concepts to strengthen teams by teaching them how to support each other, thereby enhancing their health. However, the latter is recommended as a second step once the open questions regarding incremental and criterion validity have been addressed.

Moreover, SelfCare should be integrated into health promotion interventions as an important strategy. This would involve sensitizing employees and enabling them to acknowledge their needs, providing them with offers and possibilities at work and encourage them to advocate for themselves. Initial findings demonstrate that leader and employee SelfCare can be enhanced by workplace interventions (Krick and Felfe, 2020; Vonderlin et al., 2021). Of course, this does not discharge organizations or supervisors from the responsibilities they have for their employees. Organizations and leaders have a duty to provide good working conditions, fair treatment, and health-oriented support structures, especially when considering the results highlighting the importance of StaffCare compared to PeerCare. However, employees might additionally seize the opportunity to enhance their health by seeking peer health-specific support behavior.

### 5.1. Limitations and future directions

This study has some limitations. First, data was collected in a cross-sectional design, which impairs causal interpretations. However, already the cross-sectional results revealed interesting patterns between StaffCare, LeaderCare, and PeerCare, which should be explored in more detail in future studies using longitudinal designs. This would allow to decipher indirect patterns of reciprocity in the context of health promotion that have so far only been implied. In addition to longitudinal studies, we would also recommend integrating “does not apply to my workplace” as another response option to measure PeerCare and LeaderCare. This would prevent forced choices in cases where a workplace does not offer any health promotion at all. Second, the study solely concentrated on the behavior facet of SelfCare, StaffCare, PeerCare, and LeaderCare, although the original health-oriented leadership scales also encompass the facets value and awareness. Since the scope of this paper was to examine health-specific support behavior this constriction is appropriate to the papers’ goal. However, for StaffCare it has been shown, that the aspects value and awareness make their own contribution (e.g., Santa Maria et al., 2019) and should therefore be integrated in future studies examining PeerCare and LeaderCare. In the context of health-specific support behavior awareness and value could be important pre-conditions for people to demonstrate such behavior. Third, in the scope of this study incremental validity for PeerCare above and beyond StaffCare could not be supported, questioning the unique influence of PeerCare on employee health. However, preliminary research demonstrated that the expectation of employees how much StaffCare they should ideally receive, has an impact on its effect on their health behavior (Kaluza et al., 2021). Accordingly, when the provided health-specific support behavior does not fit to the expectations, this could diminish its influence. Therefore, expectations regarding PeerCare (and StaffCare) should be integrated in future research. Another possibility might be that PeerCare influences employee health mainly, when StaffCare is limited, which could be also studied in future research. Additionally, an important prerequisite for PeerCare to be effective might be that StaffCare is stable over time. Similarly, recent research demonstrated that leader-member exchange influences recovery from work via positive effect only when its variability is low (Volmer et al., 2022). All in all, future research regarding incremental validity should include the specific conditions under which health-specific support behavior is needed and by whom to clarify the dynamics between StaffCare and PeerCare. Related to this subject is the question how health-specific support behavior and general social support are related with each other. Receiving social support has also been associated with better health status (Viswesvaran et al., 1999; Schmiedl et al., 2022), and correlations of health-specific support behavior and the regarding social support measures in this study were rather high according to Cohen’s (1988) criteria. This might be an indication that a good general support climate in the team has positive effects on health-specific support processes. To put it in other words, team members showing social support might also tend to show health-specific support behavior. Therefore, future research should examine under which conditions different support aspects positively influence each other and how they comparatively contribute to health status. This approach also provides an additional starting point for demonstrating incremental validity.

Fourth, this study only considered the perspective of the subordinates. Because the study’s focus was on employees and their role as active contributors in health-specific support behavior processes, concentration on their self-perception was appropriate to the study’s objectives. However, incorporating leaders’ assessments into future studies, particularly in relation to LeaderCare and their own health, would provide additional insights. Besides leaders, it would also be of additional interest to include entire teams instead of single persons. This would not only meet the requirements of Bakker and Demerouti (2018) to integrate higher levels in the examination of resources and demands, but would also open up a variety of possibilities from which to obtain a better understanding of the effects of health-specific support behavior processes in teams. Integrating leaders and their teams would allow the examination of the reciprocal processes of StaffCare and LeaderCare. The initial results of this study revealed the strong effect of StaffCare on LeaderCare, which can be seen as the first indicator of a reciprocal process. Longitudinal studies could provide further insights as to how reciprocal processes evolve over time and about the nature of the relationship: Are the managers the driving force and the employees only react to the manager’s behavior, or can both parties initiate this reciprocal process. Similarly, the interplay of health-specific support behavior between co-workers in the team needs further investigation concerning issues of interaction and reciprocity. Overall, several questions regarding the holistic health-specific support behavior process in the workplace remain open for future research.

## 6. Conclusion

Building on a growing body of literature about health-oriented leadership, this study offered a holistic approach of health-specific support behavior by complementing the healthy leadership style. Therefore, the measures PeerCare and LeaderCare were introduced, which capture employees’ health-specific support behavior regarding their coworkers and leaders. The results suggest strong relations between the various forms of health-specific support with employees being an integral and active part of the process. In addition, the study underscores the key role of employee SelfCare in individual health. However, the specific dynamics and interactions in this holistic model still need to be studied in more detail. This will allow for a comprehensive coverage of health-specific support processes in teams and will contribute to a better understanding of how to promote healthier workplaces.

## Data availability statement

The raw data supporting the conclusions of this article will be made available by the authors, without undue reservation.

## Ethics statement

The studies involving human participants were reviewed and approved by Ethics Committee of Faculty 2 of Technische Universität Braunschweig. The patients/participants provided their written informed consent to participate in this study.

## Author contributions

NG, E-MS, and SK contributed to conception and design of the study. NG conducted the data collection, data preparation, and preparation of the first draft of the manuscript in consultation with E-MS. NG and E-MS performed statistical analyses. All authors contributed to manuscript revision, read, and approved the submitted version.

## Conflict of interest

The authors declare that the research was conducted in the absence of any commercial or financial relationships that could be construed as a potential conflict of interest.

## Publisher’s note

All claims expressed in this article are solely those of the authors and do not necessarily represent those of their affiliated organizations, or those of the publisher, the editors and the reviewers. Any product that may be evaluated in this article, or claim that may be made by its manufacturer, is not guaranteed or endorsed by the publisher.
